# CARM1 accelerates the growth of liver cancer cells by enhancing ARAF

**DOI:** 10.1016/j.gendis.2024.101395

**Published:** 2024-08-23

**Authors:** Xiaoxue Jiang, Libo Xing, Yi Lu, Rushi Qin, Liyan Wang, Shuting Song, Xinlei Liu, Sijie Xie, Shujie Li, Dongdong Lu

**Affiliations:** a Shanghai Putuo People's Hospital, School of Life Science and Technology, Tongji University, Shanghai 200092, China; b Departments of Geriatrics, Zhongshan Hospital, Fudan University, Shanghai 200032, China

CARM1 (coactivator-associated arginine methyltransferase 1) is a type I protein arginine methyltransferase and a binding protein of the p160 coactivator family.^1^ Moreover, the research shows that the absence of CARM1 leads to impaired adipocyte differentiation^2^ and disrupts normal differentiation of embryonic T cells.^3^ In addition, other studies have confirmed that CARM1 induces the expression of pluripotent genes Oct4 and Sox2 through methylation modification of histone H3, thereby damaging embryonic stem cell differentiation.^4^ Furthermore, it can be indicated that CARM1 plays an important role in different types of tumors through various pathways.^5^ Notably, it is well known that *ARAF* (v-raf murine sarcoma 3611 viral oncogene homolog) regulates cell proliferation and differentiation abilities. In this study, it is revealed that *CARM1* affects the epigenetic modification, transcriptome, and proteome to regulate the expression of related genes in liver cancer, thus regulating cell proliferation, cell metabolism, cell cycle, and other biological processes in liver cancer cells. These results provide a valuable theoretical basis for further exploring the cellular and molecular mechanisms of *CARM1* promoting the occurrence and development of liver cancer at the cellular and molecular levels.

To investigate the effect of CARM1 on liver cancer cells, the lentiviruses rLV and rLV-CARM1 were used to infect Hep3B liver cancer cells (Fig. S1A). CARM1 was overexpressed in the rLV-CARM1 group compared with the rLV group (Fig. 1A, B). The proliferation ability, colony formation ability, the weight of transplanted tumors, and PCNA (proliferating cell nuclear antigen) positive rate were significantly increased in the rLV-CARM1 group compared with the rLV group (*P* < 0.01) (Fig. 1C–F; Fig. S1B–G) and decreased in the rLV-shRNA group compared with the rLV-shRNA CARM1 group (*P* < 0.01) (Fig. S2A–G). Furthermore, similar results were obtained in Huh7 liver cancer cells (Fig. S3A–G).

**Figure 1:**
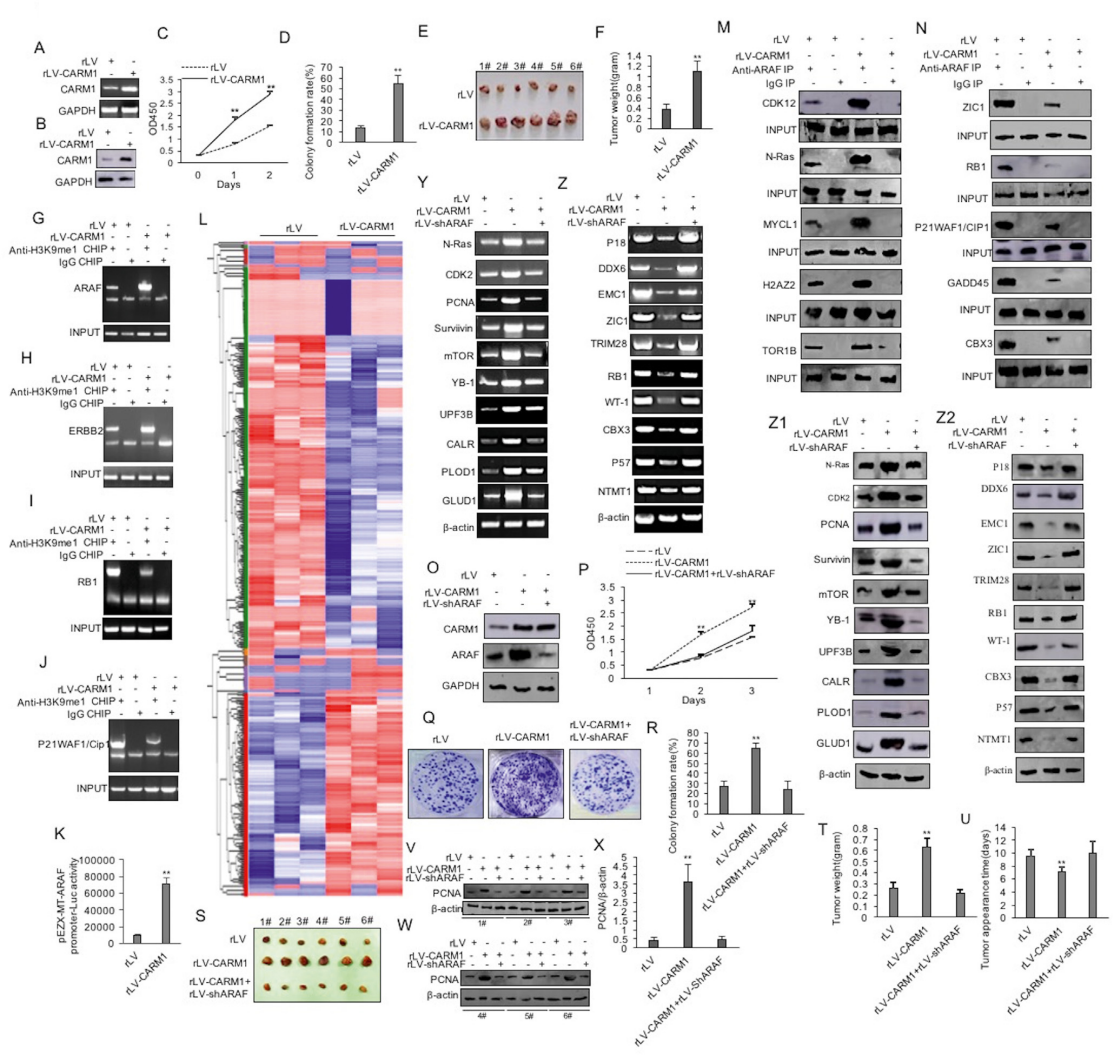
CARM1 accelerates the growth of liver cancer cells by enhancing ARAF. **(A)** CARM1 was detected by reverse transcription PCR. β-actin was used as an internal reference gene. **(B)** CARM1 was detected by western blot. β-actin was used as an internal reference gene. **(C)** CCK8 method was used to determine the cell proliferation ability. The values of each group were expressed as mean ± standard deviation (SD) (*n* = 6). ∗∗*P* < 0.01, ∗*P* < 0.05. **(D)** The colony-forming ability of cells was measured and analyzed. The values of each group were expressed as mean ± SD (*n* = 6). ∗∗*P* < 0.01, ∗*P* < 0.05. **(E)** The xenograft tumor was dissected. **(F)** Comparison of tumor size (g). The values of each group were expressed as mean ± SD (*n* = 6). ∗∗*P* < 0.01, ∗*P* < 0.05. **(G**–**J)** Chromatin immunoprecipitation sequencing with anti-H3K9me1. **(K)** The assay of ARAF promoter luciferase activity. The values of each group were expressed as mean ± SD (*n* = 3). ∗∗*P* < 0.01, ∗*P* < 0.05. **(L)** Differential protein cluster heatmap. The vertical is the clustering of samples and the horizontal is the clustering of proteins. **(M, N)** The analysis of co-immunoprecipitation with related antibodies. Western blot was used with anti-ARAF as INPUT. **(O**) ARAF was detected with western blot in the rLV group, the rLV-CARM1 group, and the rLV-CARM1+rLV-ShRNA ARAF group. β-actin was used as an internal reference gene. **(P)** CCK8 method was used to determine the cell proliferation ability. The values of each group were expressed as mean ± SD (*n* = 6). ∗∗*P* < 0.01, ∗*P* < 0.05. **(Q)** The colony-forming ability of cells was measured (photos of plate colonies). **(R)** The analysis of colony formation ability of cells. The values of each group were expressed as mean ± SD (*n* = 6). ∗∗*P* < 0.01, ∗*P* < 0.05. (**U)** The xenograft tumor was dissected for comparison of tumor size (g). **(S)** Comparison of tumor appearance time (days). The values of each group were expressed as mean ± SD (*n* = 6). ∗∗*P* < 0.01, ∗*P* < 0.05. **(T)** anti-PCNA immunohistochemical staining for obtaining PCNA positive rate (%). The values of each group were expressed as mean ± SD (*n* = 6). ∗∗*P* < 0.01, ∗*P* < 0.05. (**U–Z2)** Western blot analysis with anti-PCNA (U–X), reverse transcription PCR analysis (Y, Z), and western blot analysis (Z1, Z2) in the rLV group, the rLV-CARM1group, and the rLV-CARM1+rLV-shRNA-ARAF group. β-actin was used as an internal reference gene.

Chromatin immunoprecipitation sequencing with anti-H3K9me1 showed that the peak density distribution and the modification distribution of H3K9me1 were different between the rLV group and the rLV-CARM1 group, and there was a difference in peak binding motif in the genomic region between the rLV group and the rLV-CARM1 group, such as TCTCTCTC, GAGAGA, GAAAGAAA, CTCCTTCT, TGGTGGTG, and TGATGATG (Fig. S4A–D). In particular, the binding ability of H3K9me1 was significantly increased in the *ARAF* and *ERBB2* gene promoter regions and decreased in the *RB1* and *P21WAF1/CIp1* gene promoter regions in the rLV-CARM1 group compared with the rLV group (Fig. 1F–J). Moreover, the ARAF promoter luciferase activity was significantly increased in the rLV-CARM1 group compared with the rLV group (Fig. 1K). RNA sequencing showed that these were 77 up-regulated genes, *e.g.*, *ARAF*, *ODF2*, *GLDCP1*, *ACY1*, *AK4*, *DAPK3*, *RRPS*, *PTK2B*, *LDHAP3*, *GLDC*, *CDK2*, *PCNA*, *DMTR2*, *ASTE1*, *Survivin*, *GLUD1*, *PRNP*, *KCNIP3*, *MycL1*, *ERBB2*, *YB1*, *CDK2*, and *N-Ras*, and these were 98 down-regulated genes, *e.g.*, RTN2, WAS, SLC6A5, SEPTN3, CAVIN4, RCOR1, RIMS3, RABL2A, SLITRK1, THAP12P7, RPL36AP39, CBX3, ZBTB42, RXFP4, RB1, SH3BP1, AKT1S1, ELMO3, ZIC1, WT-1, EMC1, P21WAF1/Cip1, and P18 in the rLV-CARM1 group compared with the rLV group (Fig. S5A–F). To study the effect of CARM1 on the proteomics of human liver cancer cells, the total protein was extracted and analyzed by 10% SDS-polyacrylamide gel electrophoresis, and then the proteolytic peptides were analyzed by label-free mass spectrometry in the rLV group and the rLV-CARM1 group. The hierarchical clustering (heatmap) analysis showed that the expression of 179 proteins was up-regulated, including ARAF, N-Ras, CDK2, C-fms, MycL1, PCNA, Survivin, ERBB2, MTOR, YB1, CDK12, UPF3B, CALR, PLOD1, KATNA1, GLUD1, KATNA1, ANXA2, SERPINB1, ADK, PPP1R18, and ITGA5A, and the expression of 281 proteins was down-regulated, including G3BP2, TRIM28, DDX6, EMC1, ZIC1, P21WAF1/CIp1, P18, UBE2H, RB1, WT1, CBX3, CTNNBL1, TRIM28, H3-3B, NTMT1, RCOR1, RABL2A, EXOSC7, TUBB3, SH3BP1, and SLC6A5 (Fig. 1L; Fig. S6A–E). The results showed that CARM1 affects the interaction proteomics of the serine/threonine protein kinase ARAF and there were 100 up-regulated and 107 down-regulated ARAF interaction proteins (Fig. S7A–E). The co-immunoprecipitation with anti-ARAF showed that the interaction between ARAF and CDK12, N-Ras, MYCL1, XRCC5, H2AZ2, SMAD2, or TOR1B was significantly increased and the interaction between ARAF and ZIC1, RB1, GADD45, CBX3, P21WAF1/Cip1, OSTC, or DDX6 was significantly reduced in the rLV-CARM1 group compared with the rLV group (Fig. 1M, N). Given that CARM1 enhanced the expression of ARAF and the ARAF-interaction networks, we considered whether the cancerous functions of CARM1 were associated with ARAF. Next, we analyzed whether ARAF knockdown influenced the carcinogenic functions of CARM1 in liver cancer. As shown in Figure 1O, *CARM1* was significantly increased in the rLV-CARM1 group and the rLV-CARM1+rLV-shRNA ARAF group. Moreover, ARAF was significantly increased in the rLV-CARM1 group and decreased in the rLV-CARM1+rLV-ShRNA ARAF group compared with the rLV group. Although the proliferation ability (24 h: *P* = 0.00635; 48 h: *P* = 0.0018), the colony formation ability (27.53% ± 5.07% *vs*. 65.08% ± 4.28%; *P* = 0.0087), the weight of transplanted tumors (0.258 ± 0.056 g *vs*. 0.63 ± 0.077 g; *P* = 0.00007), and PCNA positive rate (52.46% ± 8.22% *vs*. 79.18% ± 6.05%; *P* = 0.00263) were significantly increased in the rLV-CARM1 group compared with the rLV group, such significant alterations were not found in the rLV-CARM1+rLV-shRNA ARAF group compared with the rLV group (Fig. 1P–X; Fig. S8A, B). Although the proliferation ability, the colony formation ability, the weight of transplanted tumors, and PCNA positive rate were significantly increased in rLV-CARM1 group and were significantly decreased in the rLV-shRNA ARAF group compared with the rLV group, such significant alterations were not found in the rLV-CARM1+rLV-shRNA ARAF group compared with the rLV group (Fig. S9A–G). Although the transcriptional and translational abilities of *N-Ras*, *CDK2*, *C-fms*, *MycL1*, *PCNA*, *Survivin*, *ERBB2*, *mTOR*, *YB-1*, *CDK12*, *UPF3B*, *CALR*, *PLOD1*, *KATNA1*, and *GLUD1* were significantly increased and these abilities of *G3BP2*, *TRIM28*, *DDX6*, *EMC1*, *ZIC1*, *P21WAF1/CIp1*, *P18*, *UBE2H*, *RB1*, *WT-1*, *CBX3*, *CTNNBL1*, *TRIM28*, *H3-3B*, and *NTMT1* were significantly decreased in the rLV-CARM1 group compared with the rLV group, their transcriptional and translational abilities were not significantly altered in the rLV-CARM1+rLV-shRNA ARAF group compared with the rLV group (Fig. 1Y, Z2). Collectively, these observations suggest that ARAF determines the cancerous functions of CARM1.

In conclusion, we found that *CARM1* plays a certain positive regulatory role in the occurrence and development of liver cancer dependent on *ARAF*. This provides a valuable theoretical basis for further exploring the cellular and molecular mechanisms by which CARM1 promotes the occurrence and development of liver cancer. Moreover, it suggests that CARM1 may be a potential therapeutic target for liver cancer, which is of great significance for early diagnosis and optimization of treatment strategies for liver cancer.

## Ethics declaration

All methods were carried out in accordance with the approved guidelines. All experimental protocols were approved by the Tongji University Institutional Committee. Informed consent was obtained from all subjects. The study was reviewed and approved by the China National Institutional Animal Care and Use Committee.

## Author contributions

Dongdong Lu conceived the study and participated in the study design, performance, coordination, and manuscript writing. Xiaoxue Jiang, Libo Xing, Yi Lu, Rushi Qin, Liyan Wang, Shuting Song, Xinlei Liu, Sijie Xie, and Shujie Li performed the research and prepared the figures. All authors read and approved the final manuscript.

## Conflict of interests

The authors declared no competing interests.

## Funding

This study was supported by the 10.13039/501100001809National Natural Science Foundation of China (No. 82073130).

## References

[1] Chen D., Ma H., Hong H. (1999). Regulation of transcription by a protein methyltransferase. Science.

[2] Yadav N., Cheng D., Richard S. (2008). CARM1 promotes adipocyte differentiation by coactivating PPARgamma. EMBO Rep.

[3] Li J., Zhao Z., Carter C., Ehrlich L.I.R., Bedford M.T., Richie E.R. (2013). Coactivator-associated arginine methyltransferase 1 regulates fetal hematopoiesis and thymocyte development. J Immunol.

[4] Choi S., Jo J., Seol D.W., Cha S.K., Lee J.E., Lee D.R. (2013). Regulation of pluripotency-related genes and differentiation in mouse embryonic stem cells by direct delivery of cell-penetrating peptide-conjugated CARM1 recombinant protein. Dev Reprod.

[5] Qin H., Xu J., Gong L., Jiang B., Zhao W. (2019). The long noncoding RNA ST7-AS1 promotes laryngeal squamous cell carcinoma by stabilizing CARM1. Biochem Biophys Res Commun.

