# Supplementary material for: CARM1 accelerates the growth of liver cancer cells by enhancing ARAF
Source: Genes Dis. 2024 Aug 23;12(1):101395. doi: 10.1016/j.gendis.2024.101395 (PMC11480952; doi:10.1016/j.gendis.2024.101395)
Supplement: Multimedia component 1 [file mmc1.docx]

**Supplemental Data**

**Supplemental Materials and Methods**

**Cell Lines, Lentivirus** Human liver cancer cell line (Hep3B) and Human liver cancer cell line (Huh7) were maintained in Dulbecco’s modified Eagle medium(Gibco) in a humidified atmosphere of 5% CO_2_ incubator at 37ºC. rLV, rLV-CARM1，rLV-ShRNA-ARAF were purchased from Wu Han viraltherapy Technologies Co. Ltd..

**Cell infection** Cells were infected with Lentivirus according to manufacturer's instructions. In brief, A number of wells were inoculated in a 24-well plate, and 3~5×10^4^ target cells were inoculated in each well, and the cell fusion degree was about 70% when the Lentivirus was infected. The lentivirus solution with an accurate volume was absorbed by a pipette and added to the culture medium, which was then mixed and incubated overnight in a carbon dioxide incubator (37°C, 5% CO2). After 24 hours of lentivirus addition, the virus-containing culture medium was absorbed and replaced with fresh complete culture medium, and the incubation was continued in an incubator at 37°C. After 48 hours of lentivirus addition, the GFP expression efficiency of the lentivirus carrying the GFP reporter gene could be observed by fluorescence microscopy, and the fresh complete culture medium containing the appropriate concentration of puromycin was replaced for the lentivirus carrying the puromycin resistance gene, and the stable expression cell lines were screened.

**RT-PCR** Total RNA was purified using Trizol Reagent(TIANGEN) according to manufacturer's instructions(TIANGEN). cDNA was prepared by using oligonucleotide (dT_15_), random primers, and a Thermo Reverse Transcription kit (TIANGEN). PCR analysis was performed using 2 × PCR Taq mix (TIANGEN) under the following conditions: e.g. 3-5min at 95°C followed by 35-39 cycles at 95°C for 30 s, 55°C-60°C for 30 s, and 72°C for 30sec-1 min using the PCR detection system(Bio-Rad).

**Western Blot** The logarithmically growing cells were washed twice with ice-cold phosphate-buffered saline (Hyclone) and lysed in a lysis buffer. Cells lysates were centrifuged at 12,000g for 20 minutes at 4°C after sonication on ice , and supernatants were separated. Proteins were separated on a 10% sodium dodecyl sulfate-polyacrylamide gel electrophoresis (SDS-PAGE)and transferred onto a nitrocellulose membranes(Invitrogen). The membranes were blocked in 10% dry milk-TBSTovernight at 4°C. Following three washes in Tris-HCl pH 7.5 with 0.1% Tween 20, the blots were incubated with 0.2 µg/ml of antibody(appropriate dilution) overnight at 4°C. Following three washes, membranes were then incubated with secondary antibody (horseradish peroxidase-conjugated immunoglobulin G) overnight at 4°C. Signals were visualized by enhanced chemiluminescence plus kit(Beyotime).

**Co-immunoprecipitation (IP)** The cells were lysed in 1 ml of the whole-cell extract buffer (50 mM TrisCl pH7.6, 150 mM NaCl, 0.5-1%NP40, 0.1mM EDTA and 1.0mM DTT) with protease inhibitor cocktails. In brief, five-hundred-microliter cell lysates was pre-cleared with 30μl protein G/A-plus agarose beads (Santa Cruz) by rotation for 1 hour at room temperature and the supernatant obtained after centrifugation (1,000g) for 3 minutes at 4°C. Precleared supernatants were incubated with 2 µg of antibody by rotation for 4 hours at 4°C. And the immunoprecipitates were incubated with 30μl protein A/G-plus agarose beads by rotation overnight at 4°C, and then centrifuged at 5000rpm for 5 min at 4°C. The precipitates were washed three times×10min with beads wash solution and then resuspended in 40µl 2×SDS-PAGE sample loading buffer to incubate for 10 min at 100°C. Then Western blot was performed with another related antibody indicated in Western blotting.

**Chromatin immunoprecipitation (CHIP) assay** Cells were cross-linked with 1% (v/v) formaldehyde (Sigma) for 10 min at room temperature and stopped with 125 mm glycine for 5 min. Crossed-linked cells were washed with phosphate-buffered saline, resuspended in lysis buffer, and sonicated for 8-10 min in a SONICS VibraCell to generate DNA fragments with an average size of 500-1000 bp. Chromatin extracts were diluted 5-fold with dilution buffer, pre-cleared with Protein-A/G-Sepharose beads, and immunoprecipitated with specific antibody on Protein-A/G-Sepharose beads. After washing, elution and de-cross-linking, the ChIP DNA was detected by either traditional PCR.

**Cells proliferation CCK8 Assay** The cell proliferation reagent CCK8 is purchased from Beyotime and the operation according to the manufacturer instruction. In brief, cells at a concentration 4×10^3^ were seeded into 96-well culture plates in 100μl culture medium. Before detected, add 10μg/well cell proliferation reagent CCK8(Beyotime) and incubate for 4 hours at 37°C and 5% CO_2_ . Measure the absorbance of the samples against a background control as blank using a microplate(ELISA) reader. The wave length for measuring the absorbance of the formazan product is 450nm according to the filters available for the ELISA reader. Each sample was assayed at daily intervals after seeding for up to 3 days consecutively.

**Colony-Formation Efficiency Assay** Five hundreds cells were plated in six-well plate and incubated in a humidified atmosphere of 5% CO_2_ incubator at 37ºC for 7 days . For visualization, colonies were stained with 0. 5% Crystal Violet (sigma) for more than 1 hour and then colonies were counted using a dissecting microscope by MacBiophotonics Image J.

**Xenograft transplantation *in vivo*** Four-weeks male athymic Balb/C mice were maintained in the Tongji animal facilities approved by the China Association for accreditation of laboratory animal care. Six athymic Balb/C mice per group were injected at the armpit area subcutaneously with human Hep3B suspension of 1×10^7^ in 100μl of phosphate buffered saline. The mice were observed over 4 weeks for tumor formation. Animals were stratified so that the mean tumor sizes in all groups were nearly identical. The mice were then sacrificed and the tumors recovered. The wet weight of each tumor was determined for each mouse. A portion of each tumor was fixed in 4% paraformaldehyde and embedded in paraffin for histological examination or extracted protein from the tumor tissues. There micrometers of sections(4µm) were made and stained with hematoxylin-eosin (HE) or anti-PCNA immunohistochemical staining. The use of mice for this work was reviewed and approved by the institutional animal care and use committee in accordance with China health guidelines. Student’s t-test was used to analyse *in vivo* growth patterns.

**Chip-Seq** CHIP sequencing analysis was performed according to according to the manufacturer operation manual (Novogene Co., Ltd., Beijing Nuohe Zhiyuan Technology Co., Ltd.). In brief, CHIP experiment was first performed to obtain DNA fragments bound to specific antibodies of the research target. The obtained DNA fragments were used for terminal repair, A addition, linker addition, length screening, PCR amplification, and finally high-throughput sequencing to construct a library. After the library was constructed, FastQC software was used to statistically analyze the data quality. Trimming was used to remove sequencing linker and low-quality fragments, and the pure data were obtained after pruning and filtering for subsequent analysis. Finally, FastQC software was used to statistically analyze the data quality of the pure data again. MACS2 software was used to predict the fragment size of CHIP experiment and perform subsequent peak analysis. MACS2 software was used to set qvalue<=0.05 for peak detection, and the number, width, distribution, etc. of the peaks were statistically analyzed to screen out related genes. Homer software was used to identify the genomic regions bound to the peak and show the motifs within the region. The motifs represent the sequence conservation of the peak binding site, which may be involved in regulating gene expression. The distribution of the peak relative to the gene position and the peak distribution analysis of gene functional areas were performed. The FoldEnrich value of the peak was used for difference analysis. When the FoldEnrich value was greater than 2, the difference between groups was defined as the difference peak, which could be used as a basis to obtain the difference binding sites and locate to the related genes for biological function annotation and enrichment analysis. Use the IGV browser to visually browse the bam file.

**RNA sequencing analysis** RNA sequencing analysis was performed according to according to the manufacturer operation manual (Shanghai Majorbio Bio-pharm Technology In brief, firstly, RNA was extracted from cells, and then the RNA samples were analyzed by agarose gel electrophoresis, NanoPhotometer spectrophotometer and Agilent 2100 bioanalyzer for quality control analysis; mRNA with polyA tail was enriched by Oligo(dT) magnetic beads, and then the mRNA was randomly interrupted by bivalent cations in NEB Fragmentation Buffer, and the database was built and quality checked according to the ordinary database building method or chain-specific database building method of NEB, computer sequencing (Illumina sequencing is sequencing by synthesis. Four fluorescently labeled dNTPs, DNA polymerase and connector primers were added to the flow cell for amplification. When each sequencing cluster extended the complementary chain, each fluorescently labeled dNTP was added to release the corresponding fluorescence, and the sequencing instrument captured the fluorescence signal and converted the optical signal into sequencing peak through computer software, so as to obtain the sequence information of the fragment to be tested). RNA-seq analysis includes quality control, comparison, quantification, significance analysis of differences, functional enrichment and other links. In addition, variable splicing, mutation sites, fusion gene analysis were also performed. After calculating the FPKM of all genes in each sample, the distribution of gene expression levels in different samples was shown by box plot. The original readcount was normalized, the sequencing depth was corrected, the hypothesis testing probability (pvalue) was calculated by statistical model, and the multiple hypothesis testing correction was performed to obtain the FDR (false discovery rate). The differential gene Venn diagram can show the overlap of differential genes between different comparison combinations, and the common or unique differential genes of certain comparison combinations can be screened through the Venn diagram. The clusterProfiler software was used to perform GO (Gene Ontology) function enrichment analysis, KEGG (Kyoto Encyclopedia of Genes and Genomes) pathway enrichment analysis, Reactome function enrichment analysis, DO (Disease Ontology) function enrichment analysis (a database describing the function of human genes and diseases), and DisGeNET enrichment analysis (DisGeNET database integrates human disease-related genes). The rMATS software was used for variable splicing analysis, including SE (Skipped exon), RI (Retained intron), MXE (Mutually exclusive exon), A5SS (Alternative 5 splice site 5' end exon variable splicing), A3SS (Alternative 3 splice site 3' end exon variable splicing). The GATK software was used for variant analysis of sample data, and the SnpEff software was used to annotate the variant sites. The variant sites were mainly divided into SNP and INDEL.

**Label-free Protein Mass spectrometric analysis** Mass spectrometric analysis of enzyme hydrolyzed peptides of protein without label free was performed according to according to the manufacturer operation manual (Shanghai Majorbio Bio-pharm Technology Co.,Ltd).In Brief, add appropriate protein lysate (100 mM ammonium bicarbonate, 8M urea, 0.2% SDS, pH=8), shake and mix well, and ultrasonic ice water bath for 5 min to fully cleavage. Centrifuge at 4°C and 12000 g for 15 min, take the supernatant and add the final concentration of 10 mM DT for 1 h, then add sufficient IAM, and avoid light at room temperature for 1 h. Add 4 times the volume of -20°C pre-cooled acetone at -20°C for at least 2 h, and centrifuge at 4°C and 12000 g for 15 min, collect the precipitation. Then add 1 mL -20°C pre-cooled acetone to re-suspend and wash the precipitation, and centrifuge at 4°C and 12000 g for 15 min, collect the precipitation, air dry, and add appropriate protein lysate (6 M urea, 100 mM TEAB, pH=8.5) to dissolve the protein precipitation. Then perform protein detection analysis, proteolysis, and liquid quality detection. Then, use the database software Proteome Discoverer 2.2 (PD2.2, Thermo) to search all the results. The database parameters are set as follows: the mass tolerance of the precursor ion is 10 ppm, and the mass tolerance of the fragment ion is 0.02 Da. The immobilization modification is alkylation modification of cysteine, the variable modification is oxidation modification of methionine, and the N-terminal is acetylation modification, allowing up to 2 omission sites. T-test is used to statistically analyze the quantitative results of proteins, and the quantitative differences between the experimental group and the control group are significant. The GO and IPR functional annotation (including Pfam, PRINTS, ProDom, SMART, ProSite, PANTHER databases), COG and KEGG enrichment analysis were performed using the interproscan software.

**Immunoprecipitation protein mass spectrometry** Immunoprecipitation protein mass spectrometry analysis was performed according to according to the manufacturer operation manual(Shanghai Majorbio Bio-pharm Technology Co.,Ltd). In brief，the related proteins were immunoprecipitated with related antibodies. Then, the immunoprecipitated protein were detected by Label-free Protein Mass spectrometric analysis.

**Supplemental Discussion**

CARM1 is a type I protein arginine methyltransferase and a binding protein of the p160 coactivator family [1]. Research has found that the absence of CARM1 can lead to impaired adipocyte differentiation [2,3] and disruption of normal differentiation of embryonic T cells [4].In addition, other studies have confirmed that CARM1 induces the expression of pluripotent genes Oct4 and Sox2 through methylation of histone H3, thereby damaging embryonic stem cell differentiation [5]. The studies have shown that CARM1 is involved in the occurrence and development of breast cancer cancer [6] and CARM1 activates IRE1 α/ XBP1s signaling pathway to resist endoplasmic reticulum stress and improve ovarian cancer cell survival. Several literature reports that CARM1 positively regulates the expression of transcription factor E2F1 to promote the growth of cancer cells [7]. Furthermore, CARM1 promotes the proliferation of gastric cancer cells by promoting TFE3 nuclear translocation and inducing autophagy through the cytoplasmic AMPK-mTOR pathway and the nuclear AMPK-CARM1-TFE3 pathway. Some studies have found that CARM1 inhibits MDH1 by methylation of MDH1 and destruction of its dimer, leading to the reduction of glutamine metabolism, thus making pancreatic cancer cells sensitive to oxidative stress and inhibiting cancer cell proliferation[8,9]. The methylation modification of multifunctional transcription factor Sox-2 by CARM1 promotes the progression of laryngeal squamous cell carcinoma [10]. It can be seen that CARM1 plays an important role in different types of tumors through various pathways.

It is well known that ARAF is located on the Xp11.3 of human chromosome and belongs to the one of the serine/Threonine protein kinase (RAF kinase) family, which regulates cell proliferation, differentiation, migration and survival [11-13]. Studies have found that ARAF can affect the duration of MAPK pathway activation and maintain cell cycle progression [14]. ARAF also plays an important role in the proliferation of vascular smooth muscle [15]. RNA interference mediated ARAF silencing can effectively inhibit the proliferation, colony formation, migration and invasion of gallbladder cancer cells [16]. Furthermore, ARAF mutations can trigger reactivation of the MAPK pathway, leading to drug resistance in tumor cells to RAF dimer inhibitors [17].

In this study, it is revealed that CARM1 affects the epigenetic modification, transcriptome, and proteome to regulate the expression of related genes in liver cancer, thus regulating cell proliferation, cell metabolism, cell cycle, and other biological processes in liver cancer cells. It also found that CARM1 plays a positive regulatory role in the occurrence and development of hepatoma by relying on ARAF. These results provide valuable theoretical basis for further exploring the cellular and molecular mechanisms of CARM1 promoting the occurrence and development of liver cancer at the cellular and molecular levels. Therefore, our results suggest that CARM1 may be a potential therapeutic target for liver cancer, which is of great significance for the early diagnosis and optimization of treatment strategies for human liver cancer.

In the present study, we clearly confirmed that CARM1 was closely related to the occurrence and development of human liver cancer through experiments *in vitro and in vivo* combined with high-throughput technology. Moreover, it proves that CARM1 plays a certain positive regulatory role in the hepatocarcinogenesis dependent on ARAF. Thereby, our results provide valuable theoretical basis for further studying the cellular and molecular mechanisms by which CARM1 promotes hepatocarcinogenesis at the cellular and molecular level.

In this study, we clearly demonstrate that overexpression of CARM1 significantly promotes the proliferation and colony formation ability of liver cancer cells *in vitro*, as well as the ability to form transplanted tumors in nude mice. CARM1 is an important regulatory molecule for the occurrence and development of various cancers and plays a promoting role in tumor development, which is consistent with our experimental results. On the other hand, CARM1 also plays an anti-tumor role. For example, it has been found that CARM1 regulates glycolysis through methylation of GAPDH in liver cancer [18]. It suggests that CARM1 plays different roles in different cancers.

Evidently ,Based on Chromatin immunoprecipitation sequencing (ChIP seq), It is worth mentioning that CARM1 affected the modification ability of H3K9me1 in some gene promoter regions in liver cancer cells, thus affecting the transcription level of genes. Especially, the degree of modification of H3K9me1 in the ARAF and ERBB2 promoter regions increases, thereby promoting their transcriptional ability. The degree of modification in the RB1 and P21WAF1/CIp1 promoter regions decreased, thereby inhibiting their transcription. ERBB2 protein, a member of the epidermal growth factor receptor family, is a protein Tyrosine kinase. On the one hand, the amplification of ERBB2 gene leads to the overexpression of ERBB2, which leads to the imbalance of tumor cell proliferation and promotes tumorigenesis, including breast cancer, esophageal cancer, colorectal cancer, and non-small cell lung cancer [19-22].

It is obviously good that one of the widely studied functions of RB1 is to regulate gene transcription, thus participating in DNA replication, mitosis process, and cell cycle progression [23-27]. RB1 gene mutation is also found in prostate cancer, lung cancer, breast cancer and other malignant tumors **[28-30]**. P21WAF1/CIp1 (also known as p21) is a mature cell cyclin-dependent kinase antagonist, which has been proved to play a key role in controlling the process of cell cycle **[31]**.

Importantly, our observations suggest that CARM1 changes the transcriptome and proteomics of hepatoma cells. The results of functional enrichment analysis show that differential genes are mainly enriched in pathways such as amino acid metabolism, protein translation, and immunity. Differential proteins may affect the malignant proliferation of liver cancer cells by participating in regulating biological processes such as cell metabolism and cell cycle. In particular, CARM1enhances the expression of ARAF,ODF2, GLDCP1,ACY1, AK4,DAPK3,PPR8, PTK2B,LDHAP3,GLDC, CDK2,PCNA,DMTR2,ASTE1,Survivin,GLUD1,PRNP,KCNIP3,MycL1,ERBB2,YB1,N-Ras. Studies have found that the expression of AK4 is enhanced under hypoxic conditions, thereby increasing intracellular reactive oxygen species levels to stabilize HIF-1 α Protein and induce epithelial mesenchymal transition (EMT) in non-small cell lung cancer [32]. In addition, AK4 participates in the resistance of anti-tumor drugs by regulating mitochondrial activity [33].Research has shown that that downregulation of DAPK3 is associated with tumor invasion, metastasis, and poor prognosis in primary gastric cancer[34]. Further research that DAPK3 can directly phosphorylate ULK1 and activate ULK1 dependent autophagy to inhibit gastric cancer progression [35]. The abnormal expression of PTK2B frequently occurs in human astrocytomas, and the degree of malignancy of astrocytomas is significantly correlated with the expression of PTK2B [36]. Other studies have confirmed that PTK2B is an intracellular signal transduction element that mediates the interaction between microglia and glioma cells, leading to glioma cell migration [37]. GLDC expression is significantly increased in MYCN amplified neurocytomas, which is necessary for the proliferation and tumorigenesis of neurocytoma cells [38].

Moreover. CARM1inhibits the expression of RTN2,WAS,SLC6A5,SEPTN3（SEPTIN3）,CAVIN4,RCOR1,RIMS3,RABL2A,SLITRK1,CBX3,ZBTB42,RXFP4,RB1,SH3BP1,AKT1S1,ELMO3,ZIC1,WT-1,EMC1,P21WAF1/Cip1,P18. Research suggests that CBX3 is the main subtype of heterochromatin protein 1. Its main functions include participating in gene transcriptional regulation, heterochromatin formation, DNA damage repair, cellular senescence development, and maintaining telomere and Centromere stability [39-41]. The downregulation of CBX3 also affects the p21/CDKN1A pathway, leading to G1/S phase arrest in colon cancer cells, thereby affecting cell cycle distribution [42]. RXFP4 is a homologous receptor of insulin like peptide 5 (INSL5), which can be used as a marker of colorectal cancer and breast cancer progression [43-45]. SH3 domain binding protein 1 (SH3BP1) is a Rho GTPase activating protein that plays an important role in cell motility and various human cancer invasion processes by regulating the Rac1 pathway [46]. SH3BP1 is an important angiogenic factor in hepatocellular carcinoma, promoting tumor invasion and microvascular formation through the Rac1-WAVE2 signaling pathway, thereby promoting metastasis and recurrence of hepatocellular carcinoma [47].

Obviously, these findings are noteworthy that ARAF determines the carcinogenic effect of CARM1 in liver cancer. Firstly, mass spectrometry analysis of anti-ARAF immunoprecipitation Label free protein hydrolytic peptides showed that CARM1 altered the interaction network of ARAF. Secondly, CARM1 promotes the growth *in vitro and in vivo* of liver cancer cells dependent on ARAF and alter the expression ability of some genes; Thirdly, CARM1 enhances the translation capabilities of N-Ras, CDK2, C-fms, MycL1, PCNA, Survivin, ERBB2, mTPR, YB-1, UPF3B, CALR, PLOD1, KATNA1, GLUD1 via ARAF.Fourthly, CARM1 reduces the translation ability of G3BP2, TRIM28, DDX6, EMC1, ZIC1, P21WAF1/CIp1, P18, UBE2H, RB1, WT-1, CBX3, CTNNBL1, H3-3B, and NTMT1 via ARAF.. CDK2 is highly expressed and abnormally activated in many human tumors, making it a pote,ntial target for tumor treatment [48]. HHT blocks the interaction between CDK2 and Cyclin A, leading to enhanced binding between CDK2 and Trim21, which in turn recruits autophagy machines through Trim21 to degrade CDK2 in cancer cells [49]. As a member of the Myc Gene family, MYCL1 participates in many biological processes, including cell proliferation, differentiation, apoptosis [50], and is amplified and overexpressed in some malignant tumors [51-52]. Proliferating cell nuclear antigen (PCNA) is an important factor involved in protein interaction during DNA replication and repair [53,54]. Survivin is a key member of the apoptosis inhibitory protein family [55], which regulates cell cycle [56]. ZIC1 is a tumor related gene, which has been reported in gastric cancer [57], breast cancer [58] and thyroid cancer [59]. Han et al. found that ZIC1 is related to mTOR. Over expression of ZIC1 inhibits cell proliferation by inhibiting Akt/mTOR/P70S6K pathway, reduces mitochondrial Membrane potential, and promotes breast cancer cell apoptosis [60].

In this study, we found that CARM1 plays a certain positive regulatory role in the occurrence and development of liver cancer dependent on ARAF. This provides valuable theoretical basis for further exploring the cellular and molecular mechanisms by which CARM1 promotes the occurrence and development of liver cancer. Meanwhile, this suggests that CARM1 may be a potential therapeutic target for liver cancer, which is of great significance for the early diagnosis and optimization of treatment strategies for liver cancer.

**Supplemental Reference**

1.Chen D, Ma H, Hong H. Regulation of transcription by a protein methyltransferase. Science, 1999, 284(5423): 2174-2177.

2.Yadav N, Cheng D, Richard S. CARM1 promotes adipocyte differentiation by coactivating PPARgamma. EMBO Rep, 2008, 9(2): 193-198.

3.Kim D, Lee J, Cheng D, et al. Enzymatic activity is required for the in vivo functions of CARM1. J Biol Chem, 2010, 285(2): 1147-1152.

4. Li J, Zhao Z, Carter C, et al. Coactivator-associated arginine methyltransferase 1 regulates fetal hematopoiesis and thymocyte development. J Immunol, 2013, 190(2): 597-604.

5. Choi S, Jo J, Seol D W, et al. Regulation of Pluripotency-related Genes and Differentiation in Mouse Embryonic Stem Cells by Direct Delivery of Cell-penetrating Peptide-conjugated CARM1 Recombinant Protein. Dev Reprod, 2013, 17(1): 9-16.

6. Lin J, Liu H, Fukumoto T, et al. Targeting the IRE1α/XBP1s pathway suppresses CARM1-expressing ovarian cancer. Nat Commun, 2021, 12(1): 5321.

7. Frietze S, Lupien M, Silver P A, et al. CARM1 regulates estrogen-stimulated breast cancer growth through up-regulation of E2F1. Cancer Res, 2008, 68(1): 301-306.

8.Wang Y P, Zhou W, Wang J, et al. Arginine Methylation of MDH1 by CARM1 Inhibits Glutamine Metabolism and Suppresses Pancreatic Cancer. Mol Cell, 2016, 64(4): 673-687.

9.Liu F, Ma F, Wang Y, et al. PKM2 methylation by CARM1 activates aerobic glycolysis to promote tumorigenesis. Nat Cell Biol, 2017, 19(11): 1358-1370.

10.Qin H, Xu J, Gong L, et al. The long noncoding RNA ST7-AS1 promotes laryngeal squamous cell carcinoma by stabilizing CARM1. Biochem Biophys Res Commun, 2019, 512(1): 34-40.

11.Rebocho A P, Marais R. ARAF acts as a scaffold to stabilize BRAF:CRAF heterodimers. Oncogene, 2013, 32(26): 3207-3212.

12. Poulikakos P I, Rosen N. Mutant BRAF melanomas--dependence and resistance. Cancer Cell, 2011, 19(1): 11-15.

13.Heidorn S J, Milagre C, Whittaker S, et al. Kinase-dead BRAF and oncogenic RAS cooperate to drive tumor progression through CRAF. Cell, 2010, 140(2): 209-221.

14.Su W, Mukherjee R, Yaeger R, et al. ARAF protein kinase activates RAS by antagonizing its binding to RASGAP NF1. Mol Cell, 2022, 82(13): 2443-2457.e7.

15. Cioffi C L, Garay M, Johnston J F, et al. Selective inhibition of A-Raf and C-Raf mRNA expression by antisense oligodeoxynucleotides in rat vascular smooth muscle cells: role of A-Raf and C-Raf in serum-induced proliferation. Mol Pharmacol, 1997, 51(3): 383-389.

16. Lin W, Tong C, Zhang W, et al. Silencing ARAF Suppresses the Malignant Phenotypes of Gallbladder Cancer Cells. Biomed Res Int, 2020, 2020: 3235786.

17. Yen I, Shanahan F, Lee J, et al. ARAF mutations confer resistance to the RAF inhibitor belvarafenib in melanoma. Nature, 2021, 594(7863): 418-423.

18. Zhong X Y, Yuan X M, Xu Y Y, et al. CARM1 Methylates GAPDH to Regulate Glucose Metabolism and Is Suppressed in Liver Cancer. Cell Rep, 2018, 24(12): 3207-3223.

19. Li N, Bu X, Wu P, et al. The "HER2-PI3K/Akt-FASN Axis" regulated malignant phenotype of colorectal cancer cells. Lipids, 2012, 47(4): 403-411.

20. Tarantino P, Jin Q, Tayob N, et al. Prognostic and Biologic Significance of ERBB2-Low Expression in Early-Stage Breast Cancer. JAMA Oncol, 2022, 8(8): 1177-1183.

21. Wu R, Yuan B, Li C, et al. A narrative review of advances in treatment and survival prognosis of HER2-positive malignant lung cancers. J Thorac Dis, 2021, 13(6): 3708-3720.

22. Oh D Y, Bang Y J. HER2-targeted therapies - a role beyond breast cancer. Nat Rev Clin Oncol, 2020, 17(1): 33-48.

23. Goodrich D W. The retinoblastoma tumor-suppressor gene, the exception that proves the rule. Oncogene, 2006, 25(38): 5233-5243.

24. Burkhart D L, Sage J. Cellular mechanisms of tumour suppression by the retinoblastoma gene. Nat Rev Cancer, 2008, 8(9): 671-682.

25.Sherr C J. Cell cycle control and cancer. Harvey Lect, 2000, 96: 73-92.

26. Nevins J R. The Rb/E2F pathway and cancer. Hum Mol Genet, 2001, 10(7): 699-703.

27.Cam H, Dynlacht B D. Emerging roles for E2F: beyond the G1/S transition and DNA replication. Cancer Cell, 2003, 3(4): 311-316.

28. Chai P, Luo Y, Yu J, et al. Clinical characteristics and germline mutation spectrum of RB1 in Chinese patients with retinoblastoma: A dual-center study of 145 patients. Exp Eye Res, 2021, 205: 108456.

29. Kiet N C, Khuong L T, Minh D D, et al. Spectrum of mutations in the RB1 gene in Vietnamese patients with retinoblastoma. Mol Vis, 2019, 25: 215-221.

30. Rojanaporn D, Boontawon T, Chareonsirisuthigul T, et al. Spectrum of germline RB1 mutations and clinical manifestations in retinoblastoma patients from Thailand. Mol Vis, 2018, 24: 778-788.

31.Harper J W, Adami G R, Wei N, et al. The p21 Cdk-interacting protein Cip1 is a potent inhibitor of G1 cyclin-dependent kinases. Cell, 1993, 75(4): 805-816.

32. Jan Y H, Lai T C, Yang C J, et al. Adenylate kinase 4 modulates oxidative stress and stabilizes HIF-1α to drive lung adenocarcinoma metastasis. J Hematol Oncol, 2019, 12(1): 12.

33.] Fujisawa K, Terai S, Takami T, et al. Modulation of anti-cancer drug sensitivity through the regulation of mitochondrial activity by adenylate kinase 4. J Exp Clin Cancer Res, 2016, 35: 48.

34. Bi J, Lau S H, Hu L, et al. Downregulation of ZIP kinase is associated with tumor invasion, metastasis and poor prognosis in gastric cancer. Int J Cancer, 2009, 124(7): 1587-1593.

35. Li G M, Li L, Li M Q, et al. DAPK3 inhibits gastric cancer progression via activation of ULK1-dependent autophagy. Cell Death Differ, 2021, 28(3): 952-967.

36. Gutenberg A, Brück W, Buchfelder M, et al. Expression of tyrosine kinases FAK and Pyk2 in 331 human astrocytomas. Acta Neuropathol, 2004, 108(3): 224-230.

37. Rolón-Reyes K, Kucheryavykh Y V, Cubano L A, et al. Microglia Activate Migration of Glioma Cells through a Pyk2 Intracellular Pathway. PLoS One, 2015, 10(6): e0131059.

38. Alptekin A, Ye B, Yu Y, et al. Glycine decarboxylase is a transcriptional target of MYCN required for neuroblastoma cell proliferation and tumorigenicity. Oncogene, 2019, 38(50): 7504-7520.

39. Lachner M, O'carroll D, Rea S, et al. Methylation of histone H3 lysine 9 creates a binding site for HP1 proteins. Nature, 2001, 410(6824): 116-120.

40.Bannister A J, Zegerman P, Partridge J F, et al. Selective recognition of methylated lysine 9 on histone H3 by the HP1 chromo domain. Nature, 2001, 410(6824): 120-124.

41. Akaike Y, Kuwano Y, Nishida K, et al. Homeodomain-interacting protein kinase 2 regulates DNA damage response through interacting with heterochromatin protein 1γ. Oncogene, 2015, 34(26): 3463-3473.

42. Fan Y, Li H, Liang X, et al. CBX3 promotes colon cancer cell proliferation by CDK6 kinase-independent function during cell cycle. Oncotarget, 2017, 8(12): 19934-19946.

43. Lee H, Lee S, Jeong D, et al. Ginsenoside Rh2 epigenetically regulates cell-mediated immune pathway to inhibit proliferation of MCF-7 breast cancer cells. J Ginseng Res, 2018, 42(4): 455-462.

44.Sun G, Li Y, Peng Y, et al. Identification of differentially expressed genes and biological characteristics of colorectal cancer by integrated bioinformatics analysis. J Cell Physiol, 2019, 234(9): 15215-15224.

45. Thanasupawat T, Hammje K, Adham I, et al. INSL5 is a novel marker for human enteroendocrine cells of the large intestine and neuroendocrine tumours. Oncol Rep, 2013, 29(1): 149-154.

46. Wang J, Feng Y, Chen X, et al. SH3BP1-induced Rac-Wave2 pathway activation regulates cervical cancer cell migration, invasion, and chemoresistance to cisplatin. J Cell Biochem, 2018, 119(2): 1733-1745.

47.Tao Y, Hu K, Tan F, et al. SH3-domain binding protein 1 in the tumor microenvironment promotes hepatocellular carcinoma metastasis through WAVE2 pathway. Oncotarget, 2016, 7(14): 18356-18370.

48.Tadesse S, Anshabo A T, Portman N, et al. Targeting CDK2 in cancer: challenges and opportunities for therapy. Drug Discov Today, 2020, 25(2): 406-413.

49.Zhang J, Gan Y, Li H, et al. Inhibition of the CDK2 and Cyclin A complex leads to autophagic degradation of CDK2 in cancer cells. Nat Commun, 2022, 13(1): 2835.

50. Kc W, Satpathy A T, Rapaport A S, et al. L-Myc expression by dendritic cells is required for optimal T-cell priming. Nature, 2014, 507(7491): 243-247.

51. Roussel M F, Robinson G W. Role of MYC in Medulloblastoma. Cold Spring Harb Perspect Med, 2013, 3(11).

52.Paulson K G, Lemos B D, Feng B, et al. Array-CGH reveals recurrent genomic changes in Merkel cell carcinoma including amplification of L-Myc. J Invest Dermatol, 2009, 129(6): 1547-1555.

53.De March M, De Biasio A. The dark side of the ring: role of the DNA sliding surface of PCNA. Crit Rev Biochem Mol Biol, 2017, 52(6): 663-673.

54. Prestel A, Wichmann N, Martins J M, et al. The PCNA interaction motifs revisited: thinking outside the PIP-box. Cell Mol Life Sci, 2019, 76(24): 4923-4943.

55.Ambrosini G, Adida C, Altieri D C. A novel anti-apoptosis gene, survivin, expressed in cancer and lymphoma. Nat Med, 1997, 3(8): 917-921.

56. Wheatley S P, Altieri D C. Survivin at a glance. J Cell Sci, 2019, 132(7).

57.Ge Q, Hu Y, He J, et al. Zic1 suppresses gastric cancer metastasis by regulating Wnt/β-catenin signaling and epithelial-mesenchymal transition. Faseb j, 2020, 34(2): 2161-2172.

58. Han W, Zhang C, Gao X J, et al. Clinicopathologic and Prognostic Significance of the Zinc Finger of the Cerebellum Family in Invasive Breast Cancer. J Breast Cancer, 2018, 21(1): 51-61.

59.Qiang W, Zhao Y, Yang Q, et al. ZIC1 is a putative tumor suppressor in thyroid cancer by modulating major signaling pathways and transcription factor FOXO3a. J Clin Endocrinol Metab, 2014, 99(7): E1163-72.

60. Han W, Cao F, Gao X J, et al. ZIC1 acts a tumor suppressor in breast cancer by targeting survivin. Int J Oncol, 2018, 53(3): 937-948.

**Supplemental Figure and Figure Legends

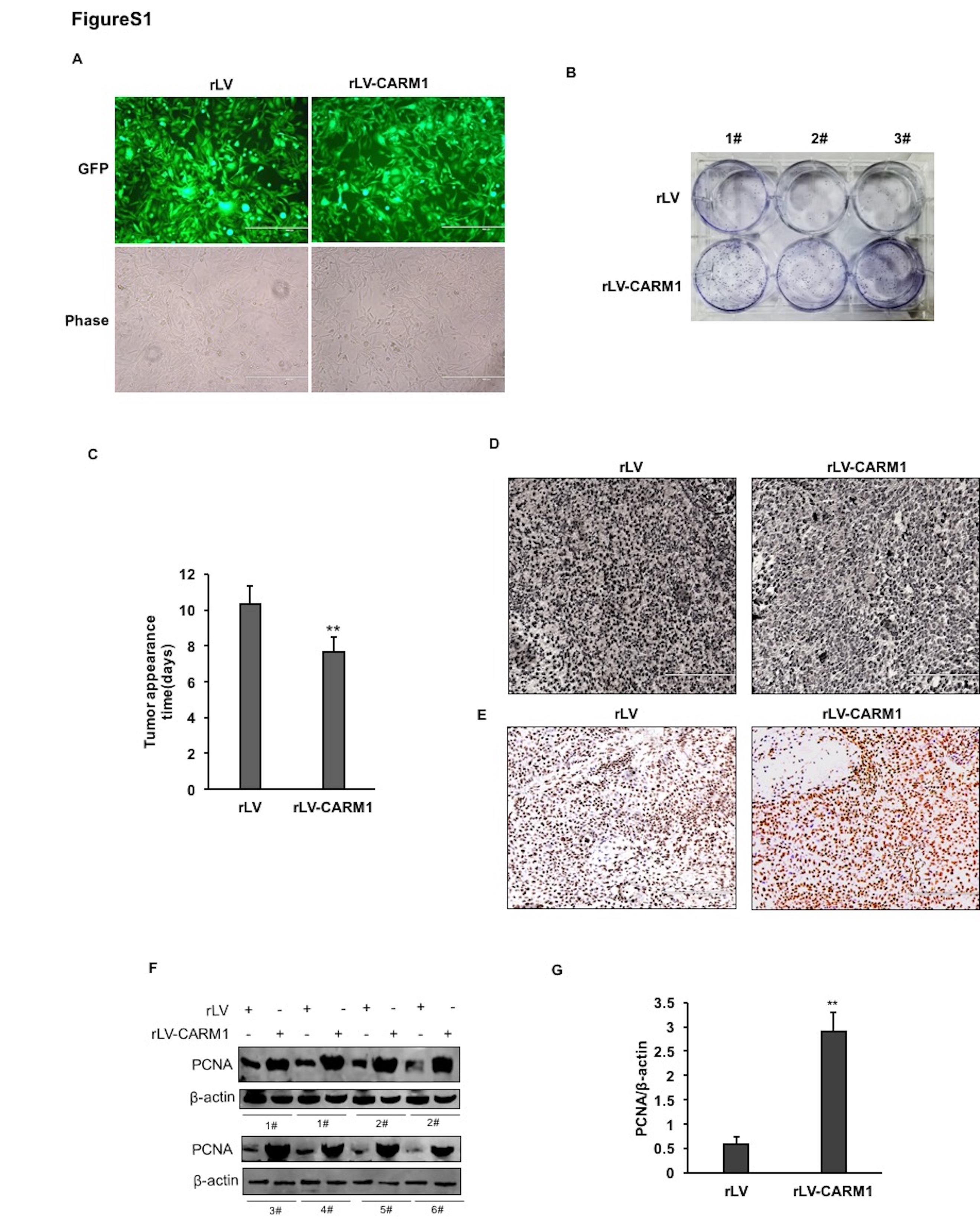
**

**Figure S1** CARM1 promotes the growth of liver cancer cells Hep3B *in vitro and in vivo*. A. Hep3B cells were infected with rLV-CARM1 and the pictures were taken under fluorescence microscope. B. The colony forming ability of cells was measured. photos of plate colonies. C. the xenograft tumor was dissected. Comparison of appearance time of xenograft tumor. The values of each group were expressed as mean ± SD (n = 6), * *, P < 0.01, and *, P < 0.05. D. The transplanted tumor tissue sections (4 μ m) fixed in 4% formaldehyde and embedded in paraffin were stained with hematoxylin eosin (HE) (original registration ×100). E. anti-PCNA immunohistochemical staining (original registration × 100). F. PCNA was detected by Western blot. β-actin was used as internal reference gene.b. G. Comparison of PCNA expression. The values of each group were expressed as mean ± SD (n=6), * *, P < 0.01, and *, P < 0.05.


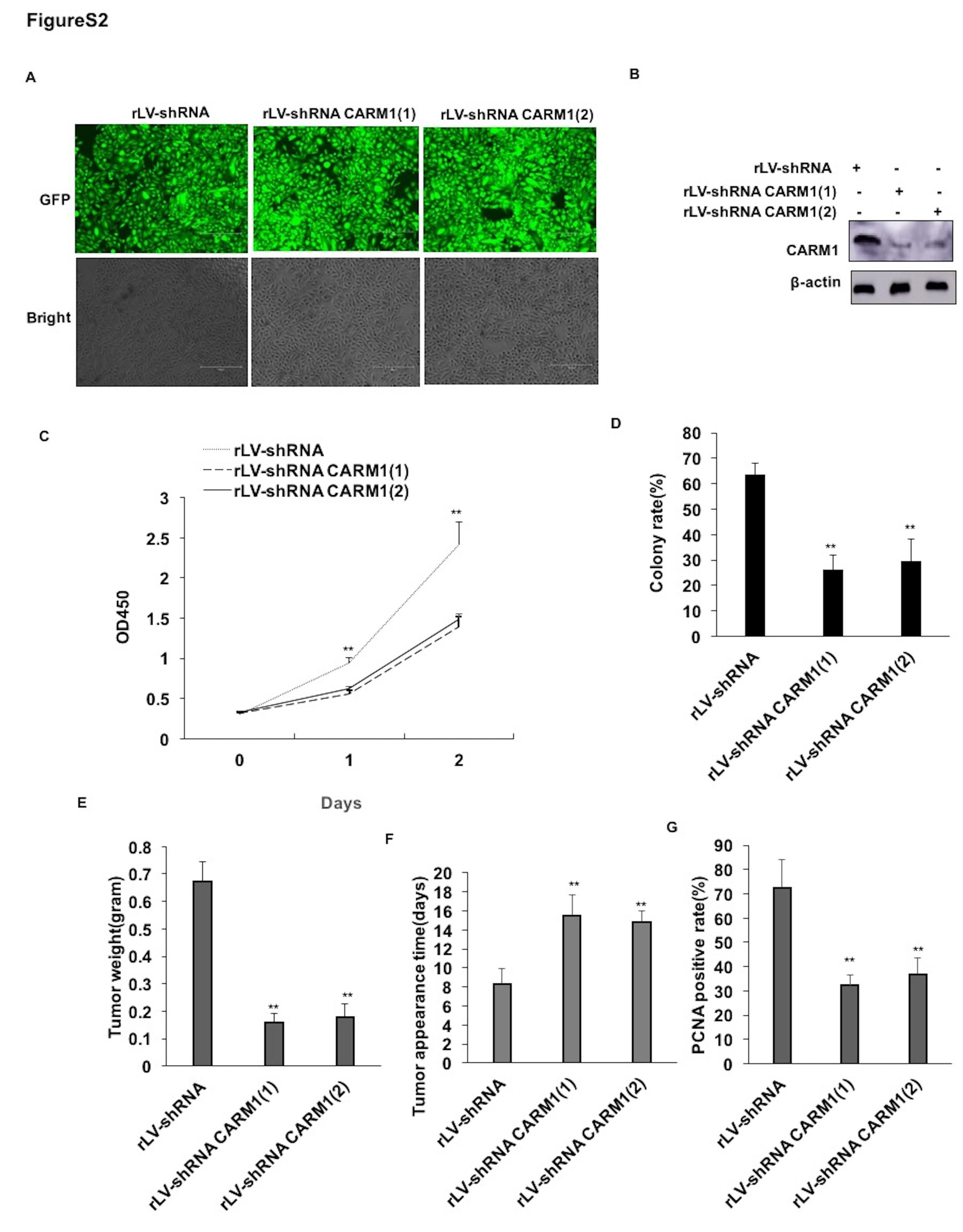


**Figure S2** CARM1 knockdown inhibits the growth of liver cancer cells Hep3B *in vitro and in vivo*. A. Hep3B cells were infected with rLV-shRNA, rLV-shRNA CARM1(1) and rLV-shRNACARM1(2) and the pictures were taken under fluorescence microscope. B. CARM1 was detected by Western blot. β-actin was used as internal reference gene. C. CCK8 method was used to determine the cell proliferation ability. The values of each group were expressed as mean ± SD (n = 3), * *, P < 0.01, and *, P < 0.05. D. The colony forming ability of cells was measured. The values of each group were expressed as mean ± standard deviation (bar ± SD, n= 6), * *, P < 0.01, *, P < 0.05. E. The xenograft tumor was dissected. Comparison of tumor size (g). The values of each group were expressed as mean ± SD (n = 6), * *, P < 0.01, and *, P < 0.05, respectively. F. the xenograft tumor was dissected. Comparison of appearance time of xenograft tumor. The values of each group were expressed as mean ± SD (n = 6), * *, P < 0.01, and *, P < 0.05. G. anti-PCNA immunohistochemical staining. PCNA positive rate(%).The values of each group were expressed as mean ± SD (n=6), * *, P < 0.01, and *, P < 0.05.

**
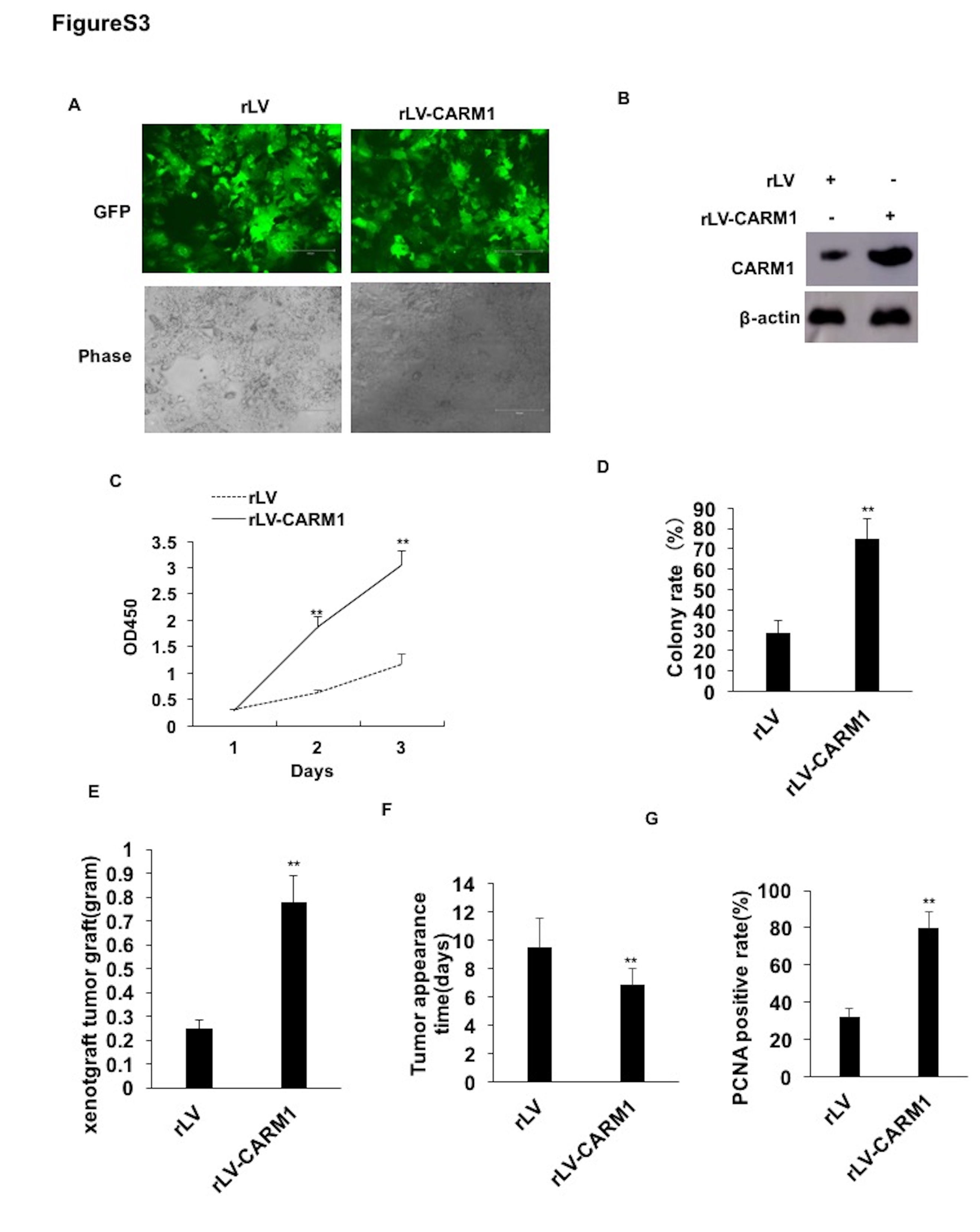
**

**Figure S3** CARM1 promotes the growth of liver cancer cells Huh7 *in vitro and in vivo*. A. Huh7 cells were infected with rLV, rLV-CARM1and the pictures were taken under fluorescence microscope. B. CARM1 was detected by Western blot. β-actin was used as internal reference gene. C. CCK8 method was used to determine the cell proliferation ability. The values of each group were expressed as mean ± SD (n = 3), * *, P < 0.01, and *, P < 0.05. D. The colony forming ability of cells was measured. The values of each group were expressed as mean ± standard deviation (bar ± SD, n= 6), * *, P < 0.01, *, P < 0.05. E. The xenograft tumor was dissected. Comparison of tumor size (g). The values of each group were expressed as mean ± SD (n = 6), * *, P < 0.01, and *, P < 0.05, respectively. F. the xenograft tumor was dissected. Comparison of appearance time of xenograft tumor. The values of each group were expressed as mean ± SD (n = 6), * *, P < 0.01, and *, P < 0.05. G. anti-PCNA immunohistochemical staining. PCNA positive rate(%).The values of each group were expressed as mean ± SD (n=6), * *, P < 0.01, and *, P < 0.05.


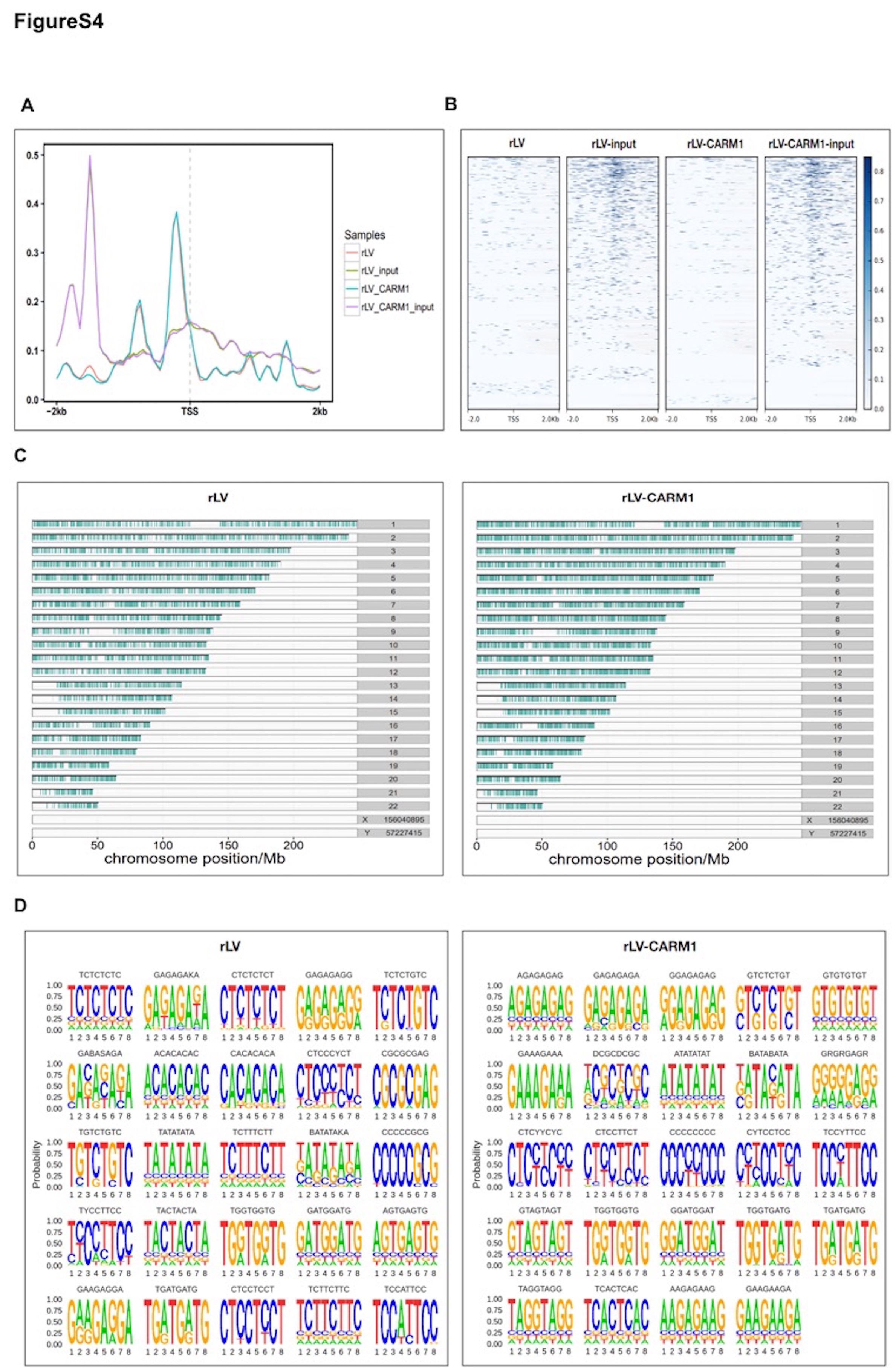


**FigureS4** Chromatin immunoprecipitation sequencing (Chip-Seq) with anti-H3K9mehigh-throughput analysis was performed in human liver cancer cells. **A-B.** The average signal distribution on the 2KB region upstream and downstream of TSS in rLV group and rLV-CARM1 group. **C.** IGV browser interface (Demo): visualization of the reads of the modification distribution of H3K9me1 on 23 pairs of chromosomes in rLV group and rLV-CARM1 group. **D.** Motif sequence.


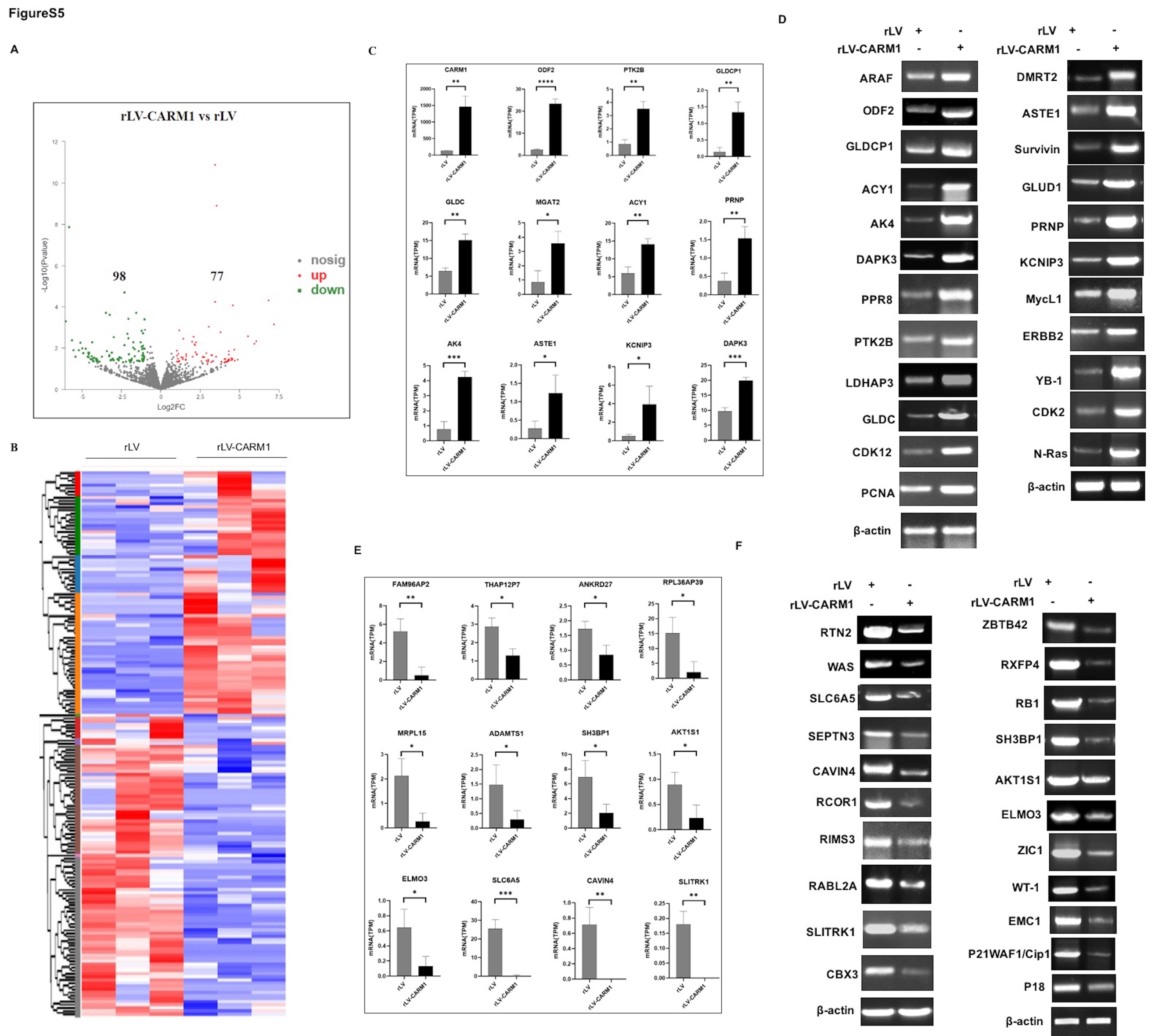


**FigureS5** CARM1 affects on the transcriptome of human liver cancer cells. **A.** The volcanic map can visually show the distribution of different genes in each comparison combination. The abscissa represents the change of gene expression multiple (log2foldchange) and the ordinate represents the significant level of gene expression difference. The up-regulated genes are indicated by red dots and the down-regulated genes are indicated by green dots. B**.** Heat map analysis (cluster) of all gene expression in the two groups. C. Up-regulated genes. D. RT-PCR analysis. β-actin was used as internal reference gene. E. Down-regulated genes. F. RT-PCR analysis. β-actin was used as internal reference gene.


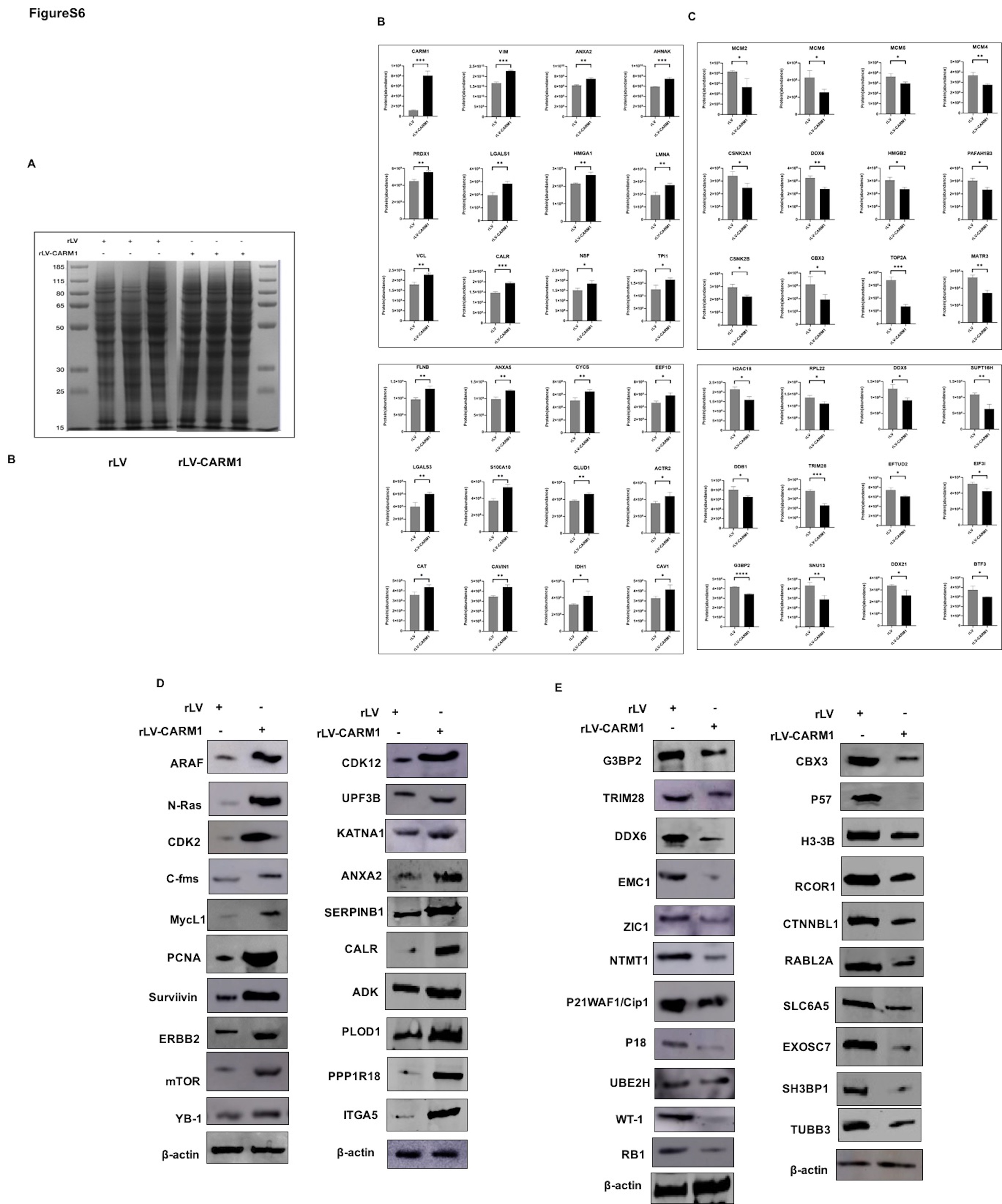


**Figure S6 CARM1 alters proteomics in liver cancer** A. The total protein was extracted and and analyzed by 10% SDS-PAGE electrophoresis. B. Histogram of up-regulated proteins . C. Histogram of down-regulated proteins. D-E. Western blot analysis. β-actin was used as internal reference gene.

**
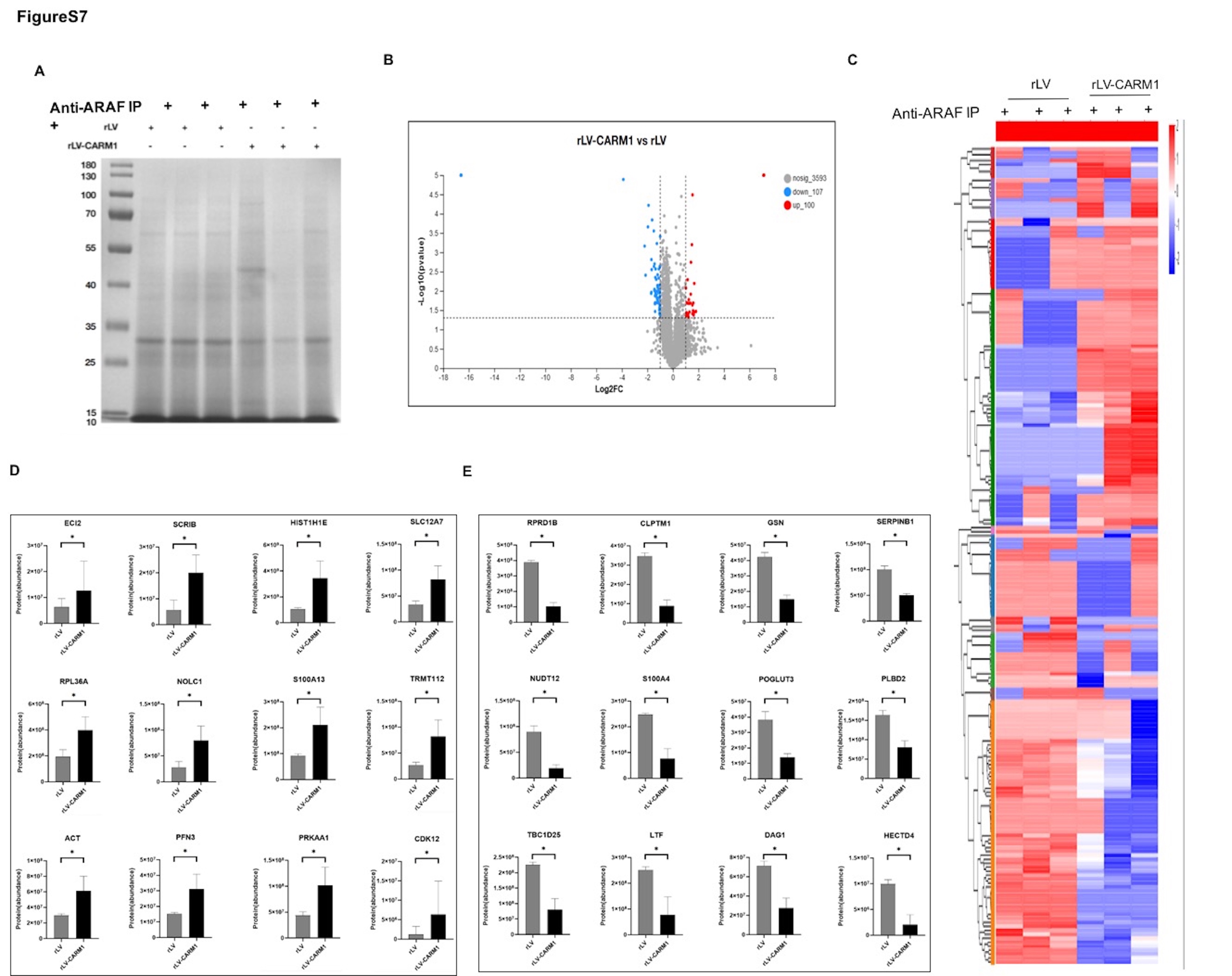
**

**FigureS7 CARM1 affect on ARAF-proteins interaction network in liver cancer**

A. The total protein was extracted and analyzed by 10% SDS-PAGE electrophoresis. B. ARAF binding differential proteins volcano map. C**.** ARAF binding differential proteins cluster Heatmap. D. the histogram diagram of ARAF binding differential proteins(up-regulaion). E. the histogram diagram of ARAF binding differential proteins(down-regulaion).


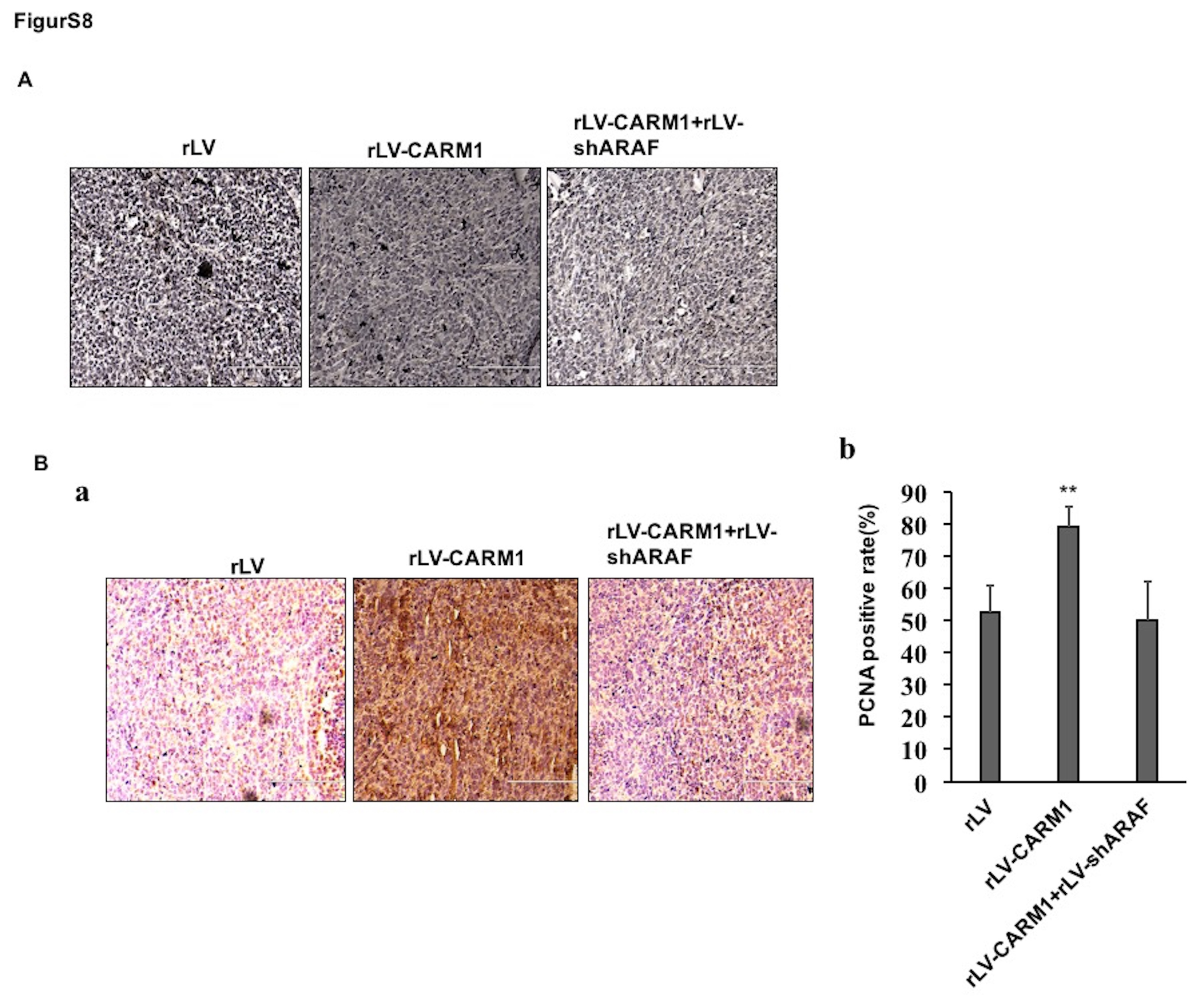


**FigureS8** CARM1 enhances the carcinogenic function dependent on ARAF. A. The transplanted tumor tissue sections (4 μ m) fixed in 4% formaldehyde and embedded in paraffin were stained with hematoxylin eosin (HE) (original registration ×100). B. a.anti-PCNA immunohistochemical staining (original registration × 100). b.PCNA positive rate(%).

**
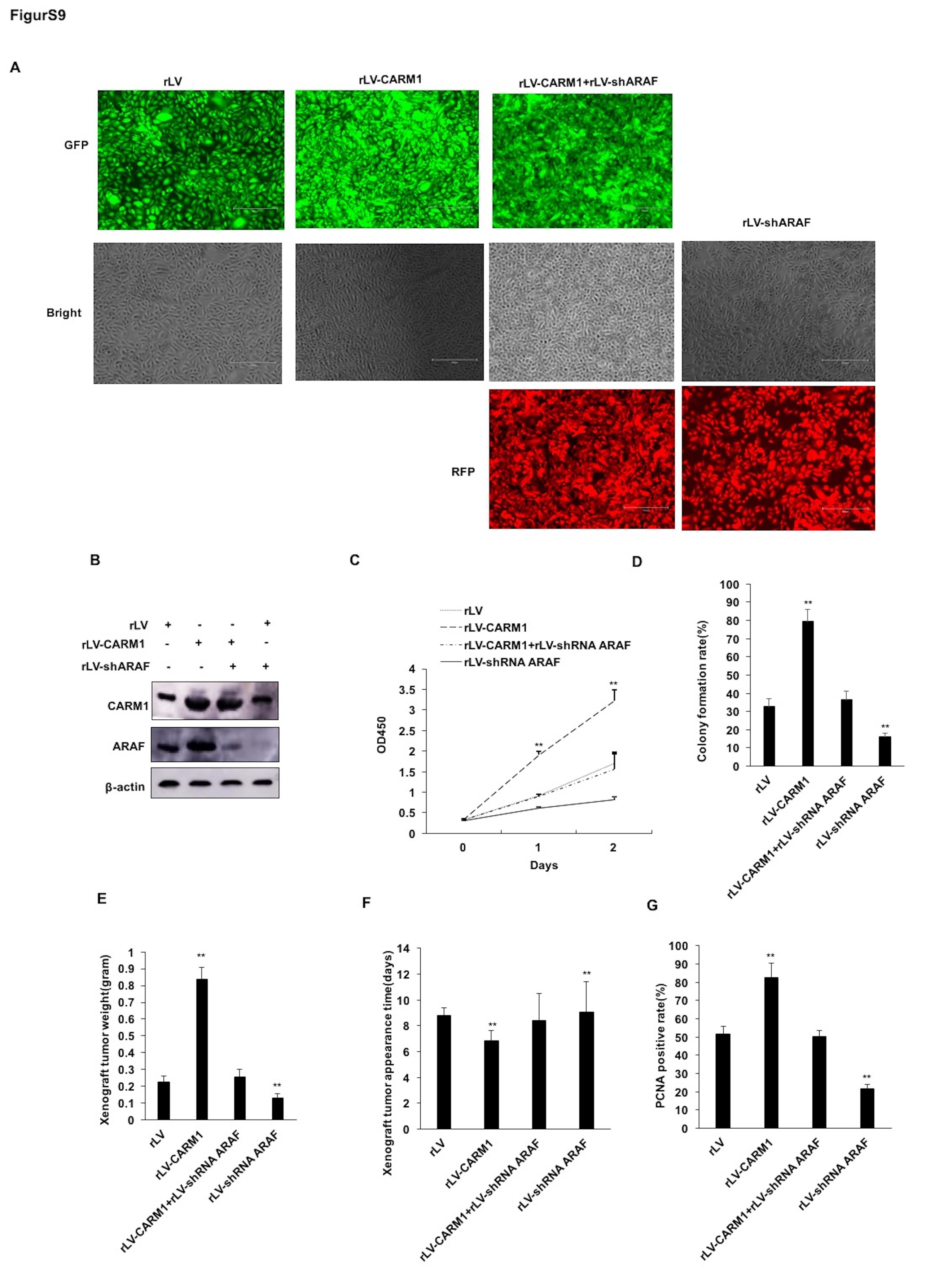
**

**FigureS9** CARM1 and ARAF affects the growth of liver cancer cells Hep3B *in vitro and in vivo*. A. Hep3B cells were infected with rLV, rLV-CARM1, rLV-CARM1+ rLV-shRNA ARAF and rLV-shRNA ARAF, and the pictures were taken under fluorescence microscope. B. CARM1 and ARAF were detected by Western blot. β-actin was used as internal reference gene. C. CCK8 method was used to determine the cell proliferation ability. The values of each group were expressed as mean ± SD (n = 6), * *, P < 0.01, and *, P < 0.05. D. The colony forming ability of cells was measured. The values of each group were expressed as mean ± standard deviation (bar ± SD, n= 6), * *, P < 0.01, *, P < 0.05. E. The xenograft tumor was dissected. Comparison of tumor size (g). The values of each group were expressed as mean ± SD (n = 6), * *, P < 0.01, and *, P < 0.05, respectively. F. the xenograft tumor was dissected. Comparison of appearance time of xenograft tumor. The values of each group were expressed as mean ± SD (n = 6), * *, P < 0.01, and *, P < 0.05. G. anti-PCNA immunohistochemical staining. PCNA positive rate(%). The values of each group were expressed as mean ± SD (n=6), * *, P < 0.01, and *, P < 0.05.
